# A New Vestibular Stimulation Mode for Motion Sickness With Emphatic Analysis of Pica

**DOI:** 10.3389/fnbeh.2022.882695

**Published:** 2022-05-04

**Authors:** Zhi-Hao Zhang, Li-Peng Liu, Yan Fang, Xiao-Cheng Wang, Wei Wang, Ying-Shing Chan, Lu Wang, Hui Li, Yun-Qing Li, Fu-Xing Zhang

**Affiliations:** ^1^Department of Human Anatomy, Histology and Embryology & K.K. Leung Brain Research Centre, School of Basic Medicine, Fourth Military Medical University, Xi’an, China; ^2^Department of Anatomy, Medical College, Yan’an University, Yan’an, China; ^3^Center of Clinical Aerospace Medicine, School of Aerospace Medicine, Fourth Military Medical University, Xi’an, China; ^4^Department of Pharmacology, Xi’an Biomedicine College, Xi’an, China; ^5^School of Biomedical Sciences, Li Ka Shing Faculty of Medicine, The University of Hong Kong, Hong Kong, Hong Kong SAR, China

**Keywords:** hypothesis testing, motion sickness, pica, rat, rotation, conditioned taste avoidance, vestibular nucleus

## Abstract

Motion sickness (MS) was frequently introduced for rodents in research work through passive motion that disturbed vestibular signals in the presence of visual and aleatory, proprioceptive inputs. Inducement of MS in this way causes conflicting signals that activate intermixed neural circuits representing multimodal stimulation. From reductionism, a lab setup to elicit rat MS via vestibular stimulation was configured in the present study for MS study in connection with dissection of the central vestibular component causally underlying MS. The individual animal was blinded to light with a custom-made restrainer, and positioned at an inclination of 30° for otolith organs to receive unusual actions by gravitoinertial vector. Following a 2-h double-axis (earth–vertical) rotation involving angular acceleration/deceleration, a suit of behaviors characterizing the MS was observed to be significantly changed including pica (eating non-nutritive substance like kaolin), conditioned taste avoidance and locomotion (*p* < 0.05). Notably, for the statistical hypothesis testing, the utility of net increased amount of kaolin consumption as independent variables in data processing was expounded. In addition, Fos-immunostained neurons in vestibular nucleus complex were significantly increased in number, suggesting the rotation-induced MS was closely related to the vestibular activation. In conclusion, our work indicated that the present setup could effectively elicit the MS by disturbing vestibular signals in rat in the context of well-controlled proprioceptive inputs and lack of visual afference.

## Introduction

Motion sickness (MS) is often experienced by human undergoing terrestrial traveling with unusual movement exposure and space traveling, or being immersed in computer-generated environment such as virtual reality (Money, 1970; Koch et al., 2018; Cohen et al., 2019; Leung and Hon, 2019). Increased salivation, pallor, nausea and/or vomiting are common symptoms that are characteristics of MS, among others (Money, 1970; Koch et al., 2018; Cohen et al., 2019; Leung and Hon, 2019). Although the MS syndrome has been known to be mediated by sensorimotor- and autonomic-reflexes that constitutively involve multiple structures such as nucleus of the solitary tract, dorsal vagus motor nucleus, and vestibular nuclear complex (VNC) (Money, 1970; Hornby, 2001; Balaban et al., 2014; Oman and Cullen, 2014; Yates et al., 2014; Zhong et al., 2021), the multiplicity of MS symptoms renders a state that mechanically explaining all aspects of the malaise is not an easy task, especially at the cellular and molecular levels. Additionally, integration of distributed forebrain structures into the neural circuits triggering and/or modulating MS compounds the difficulty (Money, 1970; Schafe and Bernstein, 1998; Hornby, 2001; Batchelor et al., 2013; Yates et al., 2014).

For the MS, understanding its neural mechanisms facilitates chemotherapeutic prophylaxis, and *vice versa*. The neural intervention with the classic anti-emetics such as scopolamine, promethazine, and amphetamine (sympathomimetics) demonstrated the involvement of histaminergic and acetycholinergic systems in the regulation of MS (Golding, 2006; Heer and Paloski, 2006; Shupak and Gordon, 2006; Schmal, 2013). In addition, the early and more recent studies on an array of potential drugs such as 5-hydroxytryptamine, transient receptor potential vanilloid-1, neurokinin and cholecystokinin regarding their anti-emetic impacts relating to the signaling mechanisms furthered the insights into neural circuit and molecular substrates underlying MS (Javid and Naylor, 2002; Golding, 2006; Machuca-Márquez et al., 2021).

The vestibular signals play a core role in generating MS (Money, 1970; Reason, 1978; Shupak and Gordon, 2006), and it is the vestibular signals at variance either with each other (otolith–canal mismatch), or with visual and/or proprioceptive motion signals (vestibular–visual/proprioceptive mismatch) that provoke MS (Reason, 1978; Bles et al., 1998; Golding, 2006; Shupak and Gordon, 2006; Oman and Cullen, 2014).Therefore, the dissection of vestibular component is of great importance to clarifying the MS neural mechanisms as a whole; thus, a rat model with vestibular stimulation that can be simultaneously employed for understanding MS-related vestibular substrates is necessitated. Currently, the lab devices used to elicit MS in animals or human engaged conflicting signals from distinct modalities (Takeda et al., 1989; Horii et al., 1993; Javid and Naylor, 2002; Cohen et al., 2003; Golding, 2006; Schmal, 2013; Balaban et al., 2014; Nooij et al., 2017, 2021; Inprasit et al., 2018; Machuca-Márquez et al., 2021) and the MS-inducement paradigms vary greatly between the research labs (Takeda et al., 1989; Horii et al., 1993; Javid and Naylor, 2002; Cohen et al., 2003; Golding, 2006; Schmal, 2013; Balaban et al., 2014; Nooij et al., 2017, 2021; Inprasit et al., 2018; Machuca-Márquez et al., 2021), including optokinetic drum (Nooij et al., 2021), Coriolis cross-coupling in rotating wheel chair (Cohen et al., 2008), and centrifugal force-producing rotators (Takeda et al., 1996; Yu et al., 2007; Diaz et al., 2015). However, an effective one producing the MS in the definite absence of visual and well-controlled proprioceptive inputs is lacking.

In the present study, we introduced a new setup and stimulating mode for effectively eliciting MS in rats, assessed by the classic proxies for nausea and/or emesis including pica (eating of non-nutritive substance) and conditioned taste avoidance (CTA) (Mitchell et al., 1977; Fox et al., 1990; Horii et al., 1993; Takeda et al., 1996; Wang et al., 2014; Inprasit et al., 2018; Nakajima, 2018; Machuca-Márquez et al., 2021). Given that pica has been more frequently used on laboratory animals as a tool for investigation into nausea/emesis condition, and even for evaluating anti-emetic drug efficiency in translational medicine, the rat MS inducible with the present provoking mode was assessed statistically by laying special emphasis on pica (Horii et al., 1993; Takeda et al., 1993, 1996; Rudd et al., 2002; Yamamoto et al., 2002, 2011; Inprasit et al., 2018; Nakajima, 2018). Along with this, an alternative to the usual pica data processing which is presumably more conforming to truth in assessing the MS was suggested.

## Materials and Methods

### Animals

Thirty-four male Sprague–Dawley rats (200 ∼ 250 g, body weight) were purchased from Animal Center of the Fourth Military Medical University (FMMU), and the experimental procedures complied with the Animal Care and Biosafety Guidelines stipulated by the FMMU’s Committee on the Animal Experimental Welfare and Ethics. Ten animals were randomly assigned for Fos-immunoreactivity (IR) on brain sections containing VNC. Twenty-four for behavior tests were randomized into two cohorts: The first (*n* = 8) was categorized as the pilot group and the second was further subdivided equally into control (pseudo-stimulation) and rotation-stimulation groups (*n* = 8, per group) for pica test only. All animals were first caged in a breeding room under a 12:12 h light–dark cycle (8:00–20:00 light on) and controlled humidity, with water and food *ad libitum*. Those for behavior tests were then separated with free access additionally to kaolin and bottled 2% sucrose solution, which were placed diametrically opposed to the front end of cage where normal food and water were deposited.

### Ethological Test Protocol

**FIGURE 1:**
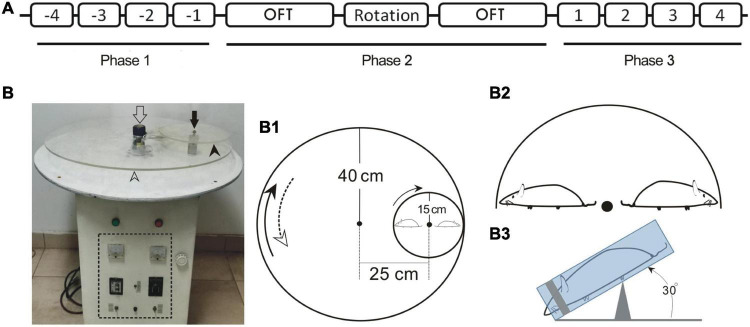
Schematic drawing depicting the timing for animal treatments in behavior test **(A)**, the prototype of MS rotator **(B)** and the construct of main mechanical working parts in association with animal positioning **(B1–B3)**. **(A)** Numbers -4 – -1 or 1–4 indicate the first to fourth full days in Phase 1 and Phase 3, respectively. **(B)** Prototype of the motor-driven double-axis rotation device for provoking MS. The open and filled arrows indicate the axes of large (open arrowhead) and small (filled arrowhead) turntables, respectively. The dashed line box demarcates the control panel area. **(B1)** Assemblage of the big and small turntables of the MS rotator. The measurements for radii and the distance between axes are denoted. **(B2)** Configuration demonstrating the radially oriented rats in relation to the small turntable. **(B3)** Lateral view of the positioning of restrained rat with anteroposterior axis tilted at an angle of 30°. OFT, open field test.

All the animals for behavior tests underwent a 3-day habituation phase prior to a three-phase procedure (Figure 1A). For pilot study (*n* = 8), a suit of behaviors including pica, CTA, and locomotion were tested, respectively. The daily kaolin intake [(KI), in (g/day)] and the consumption of 2% sucrose solution (g/day) by individuals, referring to the amount that was taken by an animal in a 24-h duration (full day), were measured for a consecutive 4 days in both Phase 1 [pre-rotation (Pre-R)] and Phase 3 [post-rotation (Post-R)]. In Phase 2, a 2-h double-axis rotation was conducted with two locomotion tests *via* open field test (OFT) intervened just before and after the rotary stimulation, respectively.

For pica-only animals (in both control and rotation groups, *n* = 8 per group), all the rats underwent the same treatments as those in pilot group but without CTA and OFT tests; and in addition, unlike rotated rats who received a 2-h rotation, the control animals experienced a pseudo-stimulation, and no actual rotation exposure (see the following discussion).

### Motion Sickness Provocation

In Phase 2, on the day immediately following the end of Phase 1, the animals in the rotation group were subjected to a 2-h double-axis rotation (16:00–18:00) in darkness with a rotary mode configured by combining a home-made rotator and self-designed setup (Figures 1A, B, B1–B3). Specifically, each rat was enclosed in a custom-made cylindrical Perspex restrainer (19-cm long with 5-cm inner diameter) (Lai et al., 2004) and cushioned posteriorly around the rump with cotton wads (Figure 1B3). A head fixer (head mask) inside the restrainer, or the sliding silicon cylinder with a coaxial cone-shape hollowed out to fit in the animal’s head was used (Lai et al., 2004). The hollow was tailored to a size such that the head fixer masked rat’s eyes and cheeks laterally while exposing the nostril through a ∼1.5 cm-diameter opening at the front, hence blocking the light. By sliding along the longitudinal groove on the restrainer’s wall, the head fixer was rightly moored where it could just restrain the animal, agreeably accommodating and immobilizing the animal’s head and body. The restrainer was then secured centrifugally on the small turntable of the home-made motor-driven rotator (see below) in a manner that the rat was disposed nose-down, head-outward with the body axis inclined at an angle of 30° (Figures 1B1–1B3).

The rotator was operated to generate a compound rotation through two different-sized, 80-cm diameter and 30-cm diameter, turntables (Figures 1B,B1), respectively, around the two earth-vertical axes, 24 cm apart. The large table ran 15 s clockwise alternating with 15 s counterclockwise with peak speed of 150°/s, whereas the small one, mounted on the large one, rotated counterclockwise at a constant speed of 450°/s (Figure 1B1). Alternation of the rotating directions of the large table produced an angular acceleration/deceleration of 90°/s^2^. This rotary stimulation session followed the end of fourth measurements of pica and CTA on the last day of Phase 1.

For control animals in Phase 2, the rats were placed just alongside the rotary equipment for 2 h (pseudo-stimulation) in phase with the rotated animals, without actual rotary stimulation.

### Measurements of Pica, Conditioned Taste Avoidance, and Food

Pica was indexed by the consumption of kaolin, which was prepared by mixing kaolin (Al_2_Si_2_O_9_H_4_; Lot #C1: 017654; Macklin Biochemical Co., Shanghai, China) and gum arabic powder in 100:1 (w/w) with distilled water, and then molded into column-shaped pellets resembling food and dried. To measure the kaolin and food intake, at 14:00-15:00 or 19:00-20:00 at the end of each of the four 24-h periods in Phase 1 or Phase 3, respectively (Figure 1A), the remaining kaolin and food including that spilled in the cage were collected and placed in an electric drying oven to be dried for 24 h; then, the daily regimen for the new round of test was arranged. The kaolin or food intake, respectively, over a 24-h time block was measured and registered. The measurements were conducted by the researchers blind to the experimental design and animal groupings. Specifically, daily consumption of kaolin or food was obtained by calculating the weight difference, in the nearest 0.01 g, between kaolin or food provided at the onset of the 24-h duration and the diet leftover (plus litter) following this time period.

In parallel with the daily KI measurements, the CTA was also examined *via* measuring the sucrose intake. Sucrose was dissolved in distilled water to make 2% solution, and daily consumption of sucrose (g/day) was recorded as the amount of solution consumed over each 24-h time block, also weighted to the accuracy of 0.01 g.

### Locomotive Ability

The locomotion was assayed through OFT both before and after the 2-h double-axis rotation exposure in Phase 2 (Figure 1A). A cubic box measuring 1.0 m × 1.0 m × 0.4 m was employed. Each animal was placed in the center of the bottom surface, and starting from 3 min later, its activities were tracked over a 15-min time course through a video recording system interfaced with a computer tracking and analyzing programs (OpenFiled, v2.8.9.7, Shanghai Mobiledatum information technology Co., Ltd, Shanghai, PR China). The primary parameters recorded included total ambulatory length (cm), and moving distance (cm) and time (s) spent in exploring the 0.5 m × 0.5 m floor central field.

### Fos Immunohistochemistry

Ten animals were assigned for Fos-IR consisted of rotation and control (pseudo-stimulation) groups (*n* = 5 for each group). The rotated and control animals experienced darkness for a 2-h rotary stimulation and pseudo-stimulation, respectively, in the same way as that of MS provocation. The animals were then sacrificed and fixed with routine immunohistochemical procedures. Briefly, anesthetization of rats *via* peritoneally introduced pentobarbital preceded fixation with 4% paraformaldehyde in phosphate buffer [(PB), pH 7.2–7.4]. The brains blocks containing VNC (Bregma: −10.08 ∼ −12.60) were cut serially in a cryostat (CM 1900, Leica, Germany) into coronal sections 25-μm thick, which were divided into four series of every four sections. One series was processed for Fos-IR.

The sections were incubated sequentially with rabbit polyclonal anti-bodies against Fos protein (1:600; Synaptic Systems), biotinylated donkey anti-rabbit IgG (1:200; Merkckmillipore), and avidin-biotin complex (1:200, VECTASTAIN ^®^ Elite ABC-HRP kit; Vectorlabs). The color reaction was conducted with 3,3′-diaminobenzidine tertrahydrochloride (DAB) as chromogen. Another series of sections was used for control immunostaining, and substituting normal rabbit serum for the primary anti-body yielded negative IR.

The digital images were captured for immunostained sections through a computer-interfaced charge-coupled device (CCD) camera system (Olympus) with fixed settings.

### Quantification and Statistics

Since VNC receives the primary afferent inputs and functionally regulates the movement and the balance, and in particular, its caudal part projects directly to brainstem structures involved in the MS development (Horowitz et al., 2005; Cai et al., 2007; Han et al., 2021), we focused on the part of VNC (Bregma-11.4 caudalward) for the neuron counting to evaluate and verify the efficacy of our rotary stimulating mode in vestibular activation. The enumeration of Fos-IR neurons in each VNC subnucleus was performed, by a researcher uninformed about the animal grouping, on the digital images from comparable brain sections of rotated and pseudo-stimulated rats. The counts were made rostro-caudally on the bilateral sides of five sections per rat, aided with the software packages, Image Pro Plus (Version 6.0.0, Media Cybernetics. Inc.) and Adobe Photoshop CS3 (Version 10.0, Adobe Systems Inc.). Only the Fos-IR nuclei with higher than background labeling intensity were taken into account. Since no difference was observed in bilateral VNCs, the data are represented as mean ± standard error of mean (SEM) per section unilaterally for each subnucleus.

For pica and CTA data analysis, only the animal who had undergone all the experimental procedures from Phase 1 throughout to the end of Phase 3 was included for MS assessment. Four “terms” employed for data processing are defined in the following. [*Note:* Herein “daily KI” refers to the KI measured and recorded at a 24-h (full-day) period].

First, “baseline KI” ($\bar{\chi }$_i/pre_) of the *i*th animal: the 4-day average of daily KI in Phase 1.

$\bar{\chi }$_i/pre_ = baseline KI for the *ithanimal* = $\frac{\sum_{k=1}^{4}\left(\chi_{i\,k}\right)}{4}$ (1), where χ_*ik*_ = daily KI (g/day) at the *k*th day in Phase 1 in relation to the *i*th animal. *k* = 1, 2, …, 4.

Second, “gross value of KI” refers to the “daily KI” measured and logged every day in Phase 1 and 3 for any individual animal. Mean of gross value is obtained by averaging the total of daily KI over specified days of duration.

Third, “net value (or gain) of KI” at a specific day in Phase 3 is defined as the net increased amount in daily KI recorded that day, or as the net amount component in daily KI added to the baseline KI. The gain in daily KI throughout Phase 3 for an individual derived its value from the Equations 1, 2.


(2) $$\text{Gain in daily KI (net value) for the }i\text{th animal = }{\bar{\chi }}_{\text{i / post}}-\text{​}{\bar{\chi }}_{\text{i / pre}}$$

$\bar{\chi }$_i/post_ = average daily KI (g/day) for the *i*th animal (in the 4-day duration) following the rotation in Phase 3.

Fourth, the mean of gain across the sampled animals in a whole group, related to Equation 2, was calculated by Equation 3:


(3) $$\text{Mean of Gain in KI per day = }\frac{\sum_{i=1}^{\text{n}}\left({\bar{\chi }}_{\text{i / post}}-\text{​}{\bar{\chi }}_{\text{i / pre}}\right)}{\text{n}}$$

$\sum_{i=1}^{\text{n}}\left({\bar{\chi }}_{\text{i / post}}-\;{\bar{\chi }}_{\text{i / pre }})=\text{ ​}({\bar{\chi }}_{\text{1 / post}}-\;{\bar{\chi }}_{\text{1 / pre }}\right)\;+\;({\bar{\chi }}_{\text{2 / post}}-\;{\bar{\chi }}_{\text{2 / pre }})\;+\;({\bar{\chi }}_{\text{3 / post}}-\;{\bar{\chi }}_{\text{3 / pre }})\;+\cdots +\;({\bar{\chi }}_{\text{i / post}}-\;{\bar{\chi }}_{\text{i / pre }}).\;i\;=\;1,2,3,\cdots ,n$ (*n*, total number of animals).

**FIGURE 2:**
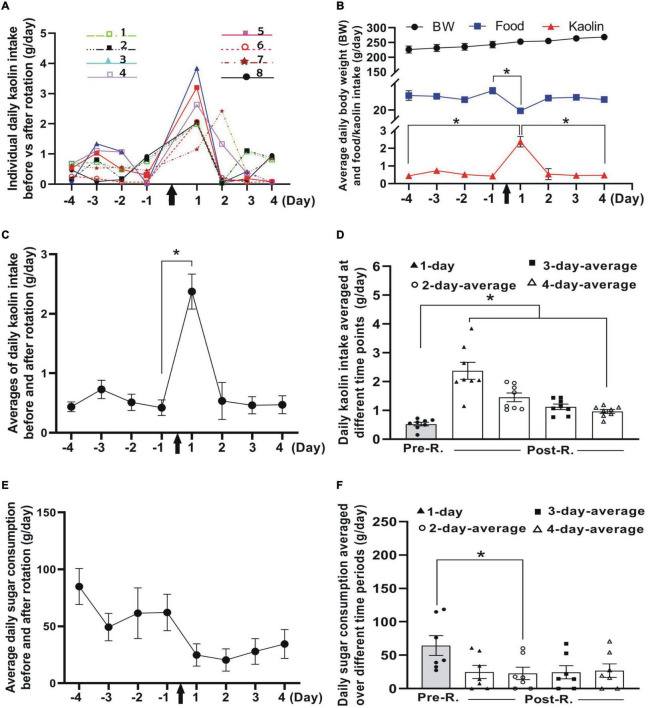
Impacts of double-axis rotation on pica and CTA in animals of pilot group (*n* = 8). **(A)** Line plot depicting the profile of daily kaolin intake (KI) (measured on 24-h basis) at different time points before and after rotation. The different colored lines and symbols represent individuals numbered for the experiment (insets). **(B)** Body weight (BW, top), food consumption (middle), and kaolin intake (lower) plotted against time points (day) before and after rotation. Note the significant decrease in food consumption at day 1 following the rotation exposure (*p < 0.05, Wilcoxon paired-test). **(C)** Mean of daily KI before and after rotation across individuals, the same data also shown in panel **(A)** (*p < 0.05, Wilcoxon paired-test). **(D)** Daily KI averaged over different duration in Phase 3 as compared with mean of baseline (Phase 1). Daily KI averaged at day 1 and over the first 2, 3 or, 4 days following the rotation are significantly higher than baseline (*p < 0.05, ANOVA *post-hoc* test). **(E)** Profile of daily sugar consumption at different days before and after rotation (*n* = 7). **(F)** Daily sugar consumption averaged over different time periods in Phase 3 as compared with mean of baseline (Phase 1) (*p < 0.05, *n* = 7; non-parametric Fridmann followed by Dunn’s test). KI, kaolin intake; Pre-R, pre-rotation; Post-R, post rotation. Black arrows indicate the time when animals were rotated.

**FIGURE 3:**
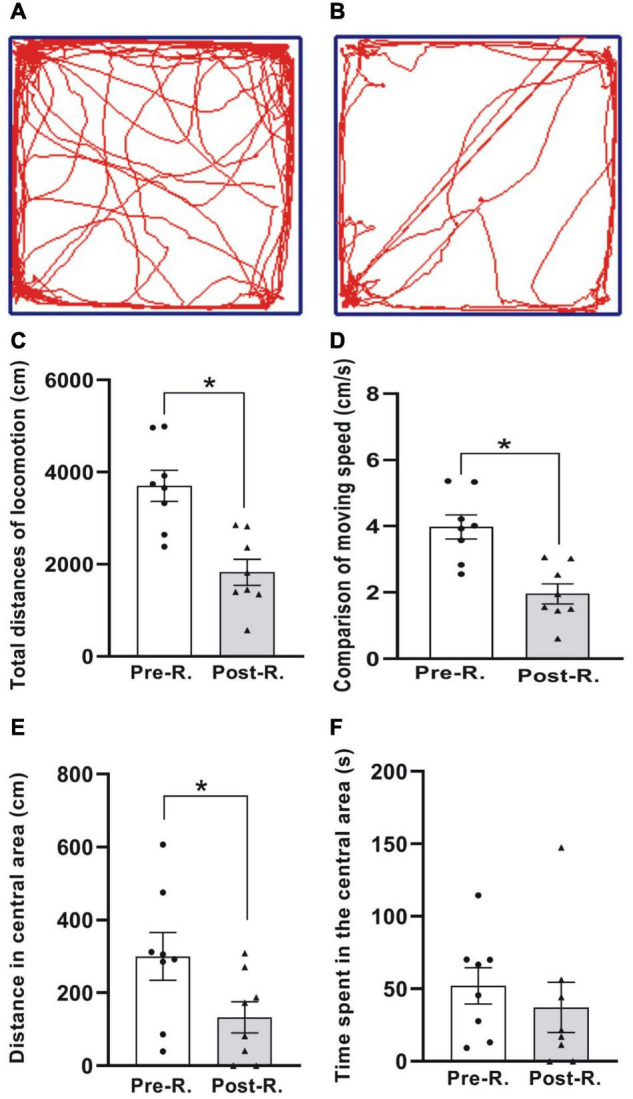
The open field test (OFT) of locomotion. **(A,B)** The digitized ambulatory traces exemplified by one animal before and after rotary stimulation, respectively. **(C–F)** Comparison of average total distance traveled **(C)**, moving speed **(D)**, moving distance **(E)**, and the time spent **(F)** in the central area for the animals between before and after rotation (*p < 0.05, *n* = 8. Wilcoxon paired-test). See legend in Figure 2 for abbreviations.

**FIGURE 4:**
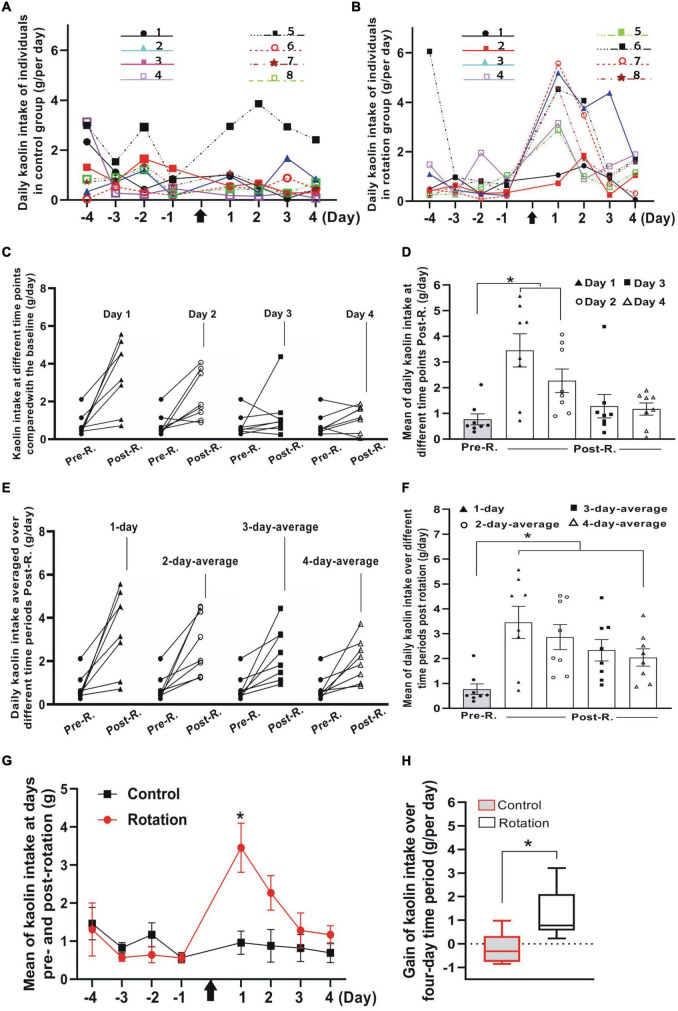
The rotary stimulation-induced pica compared between control (pseudo-stimulation) and rotation groups (*n* = 8 for each group). **(A,B)** Profiles of daily KI in Phase 1 and Phase 3 for all individuals in control **(A)** and rotation **(B)** groups. The numbered experimental animals were represented by different colored lines and symbols (inset). **(C)** The daily KI increased for the individuals after rotary stimulation. Note the differential slopes shown by the individuals in each of the four pair comparisons at different time points. Black filled circles indicate the individuals’ KI baseline. **(D)** Means of daily KI at day 1 to day 4. Black filled circles indicate the individuals’ KI baseline (*p < 0.05, ANOVA with *post-hoc* test). **(E)** Average daily KI of each individual over 1-, 2-, 3-, or 4-day time periods after rotation was compared with its baseline (black filled circles), respectively. Note the interindividual difference in average daily KI represented by the individual slopes in each of the four-pair comparisons. **(F)** Mean of average daily KI over 1-, 2-, 3-, or 4-days following the rotation compared with mean of baseline (black filled circles) (*p < 0.05, ANOVA with *post-hoc* test). **(G)** Profiles of daily KI over time before and after rotation for control and rotation groups (*p < 0.05, ANOVA with *post*-*hoc test*). **(H)** Comparison between control and rotation groups of gain in KI per day (g/day) over a four-day period following the rotation (*p < 0.05, Mann–Whitney *U*-test). Black arrows indicate the time point when animals were rotated. See legend in Figure 2 for abbreviations.

**FIGURE 5:**
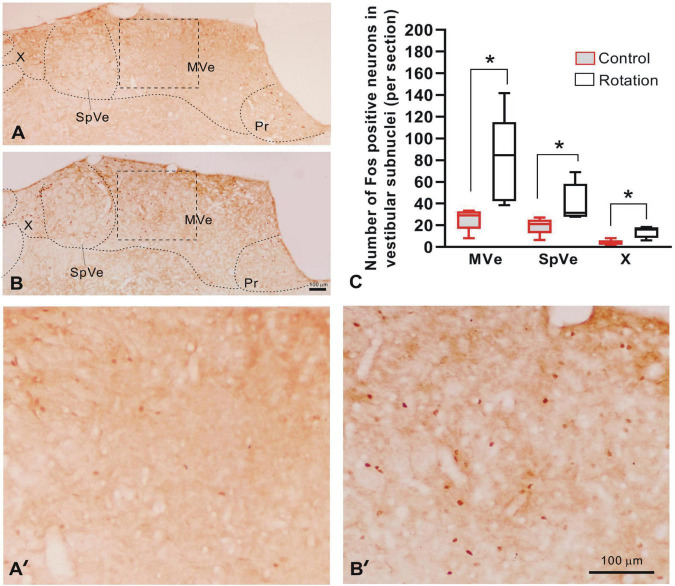
The Fos-IR in vestibular nucleus complex as compared between control (pseudo-stimulation) and rotated rats. **(A,B)** The digital images of brain sections from control and rotated rats, respectively, showing Fos-IR in vestibular subnuclei MVe, SpVe, and X. **(A’,B’)** Amplification of the rectangular areas (MVe) in panels **(A,B)** delimited by dashed lines, respectively. **(C)** The graph showing the increased number of the Fos-IR neurons per section in unilateral MVe, SpVe, and X of rotated rats, as compared with the control (*p < 0.05, *n* = 5, Mann–Whitney *U*-test). Bars = 100 μm in panels **(B,B’)**. MVe, medial vestibular nucleus; Pr, prepositus nucleus; SpVe, spinal vestibular nucleus; X, X nucleus.

The average values relating to pica, CTA or OFT are represented as mean ± SEM. The hypothesis tests were performed through GraphPad Prism 8.0.2. (GraphPad Software, Inc.). To evaluate behavior changes (pica and CTA), one-way analysis of variance (ANOVA) with *post hoc* tests was employed unless otherwise stated. With the ANOVA, “repeated measures” was designated for comparison between Pre-R and Post-R phases (Figures 2D,E, 4D,F) and “non-paired groups” for the comparison between the pseudo- and rotation-stimulated groups (Figure 4G). In addition, Mann-Whitney *U*-tests were applied to comparison between pseudo-stimulation and rotation groups (Figures 4H, 5C), and Wilcoxon paired-test for comparison of food change between Pre-R and Post-R phases (Figures 2B,C, 3C–F). the significant difference was identified if *p* < 0.05.

## Results

### Behaviors in Pilot Study

To appraise in general the effectiveness of the present stimulating mode in provoking MS, a suit of behavior phenotypes classically used as surrogates for MS including CTA, pica and locomotion was examined (Figures 2, 3). Additionally, the daily food intake and the body weight were also tracked concurrently as cues to the animals’ dietary change and state of physical health. Throughout the Phases 1–3 (Figure 1), the body weight increased over time and at the first day post rotation exposure, a significant drop in food intake (hypophagia) was manifested.

#### Pica Behavior

Following double-axis rotation, the daily KI (g/day) increased significantly from its baseline, peaked at day 1 in Phase 3 and gradually decreased to baseline level at days 3 and 4 (Figures 2A–D). Resonating with this response, a significant decrease in food ingestion following the rotation was seen at day 1 (Figure 2B). The means of daily KI following the rotation, calculated by averaging the total daily KI over different time periods (i.e., 1, 2, 3, or 4 days in Phase 3), all showed a significant increase from the mean of baseline value (0.53 ± 0.06 g/day) across individuals (*p* < 0.05, *n* = 8) (Figure 2D). The means of daily KI at first day (day 1) and in the first 2-, 3- or 4-day periods were reckoned to be 2.37 ± 0.30, 1.45 ± 0.15, 1.12 ± 0.09, and 0.96 ± 0.07, respectively (Figure 2D).

#### Conditioned Taste Avoidance

The rats demonstrated an obvious tendency to decline in the amount of sucrose solution consumption at day 1 and day 2 following the rotation, and thereafter progressively returned to the baseline level (Figures 2E,F). Compared to the baseline (64.48 ± 14.77 g/day), the first 2-day average of sugar intake in Phase 3 showed significant decrease (*p* < 0.05, *n* = 7, non-parametric Fridmann followed by Dunn’s test). The means of the sugar consumption per day (g/day) following the rotation averaged 24.86 ± 9.80, 22.66 ± 9.31, 24.42 ± 9.78, and 26.93 ± 10.08 at day 1, and over first 2, 3, and 4 days, respectively (Figure 2F).

#### Locomotion Test

To see whether the double-axis rotation impacted the animal’s moving ability, the locomotion was tested through OFT (Figure 3). The total distances (cm) traveled in the whole arena were 3704.62 ± 335.99 and 1826.93 ± 282.07 for Pre-R and Post-R sessions, respectively, showing a significant decrease following the rotation exposure (*p* < 0.05, *n* = 8) (Figure 3C); Similarly, the significant decrease in moving distance was also observed in the central area, which was shown to be 300.58 ± 65.35 cm and 132.88 ± 42.46 cm for Pre-R and Post-R, respectively (*p* < 0.05, *n* = 8) (Figure 3E). In relation to the total distances traveled, the moving speeds (cm/s) were observed to be 3.98 ± 0.36 and 1.96 ± 0.30 in Pre-R and Post-R sessions, respectively; demonstrating a significant difference (*p* < 0.05, *n* = 8). In contrast, the time (s) spent in central area showed no statistical difference, with the values recorded to be 52.15 ± 12.52 and 37.25 ± 17.26 for Pre-R and Post-R sessions, respectively (Figure 3F).

### The *de novo* Review of Pica in Paired Animal Groups

Since pica has long since been the most frequently used index for rat MS (Mitchell et al., 1977; Takeda et al., 1989; Santucci et al., 2000; Schmal, 2013; Idoux et al., 2018; Inprasit et al., 2018; Nakajima, 2018), we further examined the utility of the present stimulating mode in eliciting the rat MS by focusing specially on pica behavior test, implementing the experiments in parallel on paired animals from control (pseudo-stimulation) and rotation groups, respectively. In “Phase 1” when animals continued a normal life after habituation period, albeit some individuals in both groups ingested little or nearly no kaolin, others consumed a variable, relatively large amount (Figures 4A,B and Tables 1, 2). Such was still the case for control animals following pseudo-stimulation in Phase 3 (Figure 4A and Tables 1, 2). In contrast, in “Phase 3,” each animal in rotation group showed an increase in daily KI after rotation exposure, peaking at day 1 and declining over time (Figures 4B–D and Tables 1, 2). This pica behavior was in consistence with the finding of the foregoing pilot study (Figure 2A).

**TABLE 1:** Daily kaolin intake (KI) (g/day) of rats in control (pseudo-stimulation) and rotation groups, measured on a 24-h basis for 4 consecutive days in either Pre-R (Phase 1) or Post-R (Phase 3) sessions (mean ± SEM).

	Pre-R (Phase 1)				Post-R (Phase 3)			
		
	Day 1	Day 2	Day 3	Day 4	Day 1	Day 2	Day 3	Day 4
Control	1.46 ± 0.43	0.83 ± 0.13	1.17 ± 0.31	0.57 ± 0.14	0.96 ± 0.31	0.88 ± 0.43	0.82 ± 0.36	0.70 ± 0.26
Rotation	1.31 ± 0.70	0.57 ± 0.10	0.64 ± 0.21	0.56 ± 0.12	3.46 ± 0.65	2.27 ± 0.46	1.28 ± 0.46	1.17 ± 0.24

**TABLE 2:** Baseline (Phase 1) and average daily kaolin intake (KI) (g/day) over different time periods (Phase 3).

Animals	Pre-R (Phase 1)	Post-R (Phase 3)				
		
	Baseline level	First-day average	First 2-day average	First 3-day average	Four-day average	Gain^a^
Control. 1	1.18	1	0.77	0.53	0.5	−0.68
2	0.59	0.95	0.63	0.97	0.94	0.35
3	1.24	0.55	0.61	0.5	0.45	−0.79
4	1.02	0.17	0.16	0.2	0.17	−0.85
5	2.06	2.95	3.41	3.25	3.04	0.98
6	0.27	0.54	0.48	0.61	0.57	0.3
7	0.8	0.44	0.43	0.37	0.46	−0.34
8	0.88	1.09	0.88	0.66	0.59	−0.29
$\bar{\chi }$ ± S.E.M.	1.00 ± 0.19	0.96 ± 0.31	0.92 ± 0.36	0.89 ± 0.35	0.84 ± 0.32	−0.17 ± 0.23
Rotation. 1	0.64	1.05	1.24	1.13	0.87	0.23
2	0.43	0.72	1.28	0.94	0.96	0.53
3	0.52	5.19	4.48	4.44	3.73	3.21
4#	1.15	3.15	2.02	1.82	1.84	0.69
5	0.54	2.87	1.94	1.48	1.4	0.86
6#	2.12	4.53	4.3	3.21	2.83	0.71
7#	0.28	5.57	4.53	3.25	2.52	2.24
8	0.49	4.56	3.13	2.4	2.21	1.72
$\bar{\chi }$ ± S.E.M.	0.77 ± 0.21	3.46 ± 0.65	2.87 ± 0.50	2.33 ± 0.43	2.05 ± 0.35	1.27 ± 0.36

*Values for groups are represented as mean ($\bar{\chi }$) ± SEM.*

*^a^Average gain of KI per day (g/per day) over a 4-day period following the rotation, obtained from the Equation 2 in the text.*

To see the degree to which each individual exhibited pica and how it changed temporally, the rotation-induced daily KI at single day in Phase 3 was compared with its Pre-R KI baseline in Phase 1. The daily KI at either day 1 or day 2 following the rotation was shown to be significantly increased (*p* < 0.05, *n* = 8), contrasting with that at day 3 or day 4 (Figures 4C, D and Table 2). In addition, it was obvious that the slopes representing KI increase were different between the individuals, as could be seen in any of the four-pair comparisons between the Post-R and Pre-R (Figure 4C). When comparison was performed between pseudo- (control) and rotary-stimulation groups, the mean of daily KI at day 1 in Post-R session was significantly higher in the rotation group than that in the pseudo-stimulation group (Figure 4G and Table 1).

Given that some rats still ate relatively more amount of kaolin after day 1 following the rotation, it is reasonable to infer that using the average daily KI over longer time periods rather than a single day might be more truthful in characterizing pica behavior. Therefore, the different time points in Phase 3 were chosen to analyze the kaolin consumption for rotary-stimulation group. The results showed that for each rat following the rotation, 1-, 2-, 3-, or 4-day average of daily KI in Phase 3, all showed an increase when compared with its corresponding baseline (Phase 1) (Figure 4E and Table 2); however, the degree to which the animal increased KI differed between individuals, as shown by the differential slopes representing KI alteration (Figure 4E). At the group level, all 1-, 2-, 3- or 4-day average of daily KI in Phase 3 showed a significant difference when compared with baseline (Figure 4F and Table 2).

Since gain in KI, the resultant net increase of KI due to the rotation exposure, is more representative of MS severity than the gross amount, gain in KI per day for each animal over 4-day time block in Phase 3 was calculated, and then the means of gain per day (g/day) for the group obtained. The result showed that when compared between the pseudo- (control) and the rotary-stimulation groups, the average gain over 4-day in the rotary-stimulation group (1.27 ± 0.36, g/day) was significantly different from that in pseudo-stimulation group (−0.17 ± 0.23, g/per day) (*p* < 0.05, *n* = 8) (Figure 4H). Similarly, the significant difference between the pseudo-stimulation and the rotation groups was also observed when the gain in KI per day over either 1-, 2- or 3-day period following the rotation was compared between two groups (data not shown).

### Fos Protein Immunoreactivity in Vestibular Nuclear Complex

Following the rotary stimulation, the entire VNC exhibited Fos-IR, as represented by the brown round- or oval-shaped nuclei. In caudal part of VNC (Bregma-11.4 caudalward), rotation induced a significant increase in the number of Fos-labeled neurons when compared with control rats in terms of cell counts per section in the medial vestibular (MVe), spinal vestibular (SpVe), and X nuclei (Figures 5A–C, A’, B’). In the rotation group, the Fos-IR neurons were estimated to be 79.79 ± 18.38, 40.66 ± 7.91, and 13.81 ± 2.34 for MVe, SpVe, and X subnuclei, respectively; while in the control (pseudo-stimulation) rats, the values were 25.63 ± 4.61, 19.30 ± 3.46, and 3.90 ± 1.08 for the same nuclei, respectively.

## Discussion

We, in this study, configured a novel mode of inducing the MS in rodents and, for the first time, proposed that the “net value” be alternatively used as an independent variable in data processing for assaying MS.

### Validity of Double-Axis Rotation in Motion Sickness Inducement

Compelling evidence supports that CTA/pica are closely associated with nausea/emesis (Mitchell et al., 1977; Takeda et al., 1989, 1996; Fox et al., 1990; Horii et al., 1993; Schmal, 2013; Wang et al., 2014; Inprasit et al., 2018; Nakajima, 2018; Machuca-Márquez et al., 2021). For instance, the rats manifested and withdrew CTA/pica responses to cisplatin (a chemotherapy drug for cancer) and Ondansetron (a 5-HT3 receptor antagonist), respectively. These are also the drugs showing emetogenic and anti-emetic effects, respectively, in emetic species including human (Andrews and Horn, 2006). Additionally, under the cisplatin-induced toxicosis, pica, and emesis shared very similar neuropharmacological properties in the presence of 5-HT3 receptor antagonist (Andrews and Horn, 2006).Therefore, pica and CTA have long been considered and used as proxies for nausea and/or emesis in animals that do not vomit (Mitchell et al., 1977; Santucci et al., 2000, 2009; Yu et al., 2007; Idoux et al., 2018; Inprasit et al., 2018; Nakajima, 2018).

The present behavior results from two batches of experiments, i.e., the pilot and the paired-group (pseudo- *vs.* rotation-stimulation) studies, consistently suggested that the present MS-inducing rotary machinery was effective in eliciting MS in rats, as was suggested by the behavior change following rotary stimulation. Specifically, MS was statistically verified *via* dual ways of arithmetic data processing for pica, one by taking gross amount and the other net amount (gain) of daily KI as the variables in hypothesis testing. In agreement with pica behavior, CTA test also collaterally validated the occurrence of MS. Since the animals still ate relatively stable amount of chow (except at day 1 Post-R) and showed no abnormality in the body weight, the pica, and the CTA changes following the rotation could reasonably and causally be attributed to rotation-induced MS rather than the alimentary system illness, or the weak locomotive ability (hypolocomotion) (Figure 3). In addition, the increased Fos-IR in VNC suggested the presently elicited MS was the rats’ neural response to rotation exposure and thereby the vestibular disturbance.

### Technical Considerations

Since the functional integrity of vestibular apparatus is essential to MS (Money, 1970; Schmal, 2013), several theories were formulated, pivoting on vestibular signals, to etiologically explain the MS. These include sensory mismatch theory (Money, 1970; Schmal, 2013; Bertolini and Straumann, 2016), postural instability theory (Keshavarz et al., 2015), subjective vertical conflict theory in relation to velocity storage (Bles, 1998; Cohen et al., 2003, 2008; Bertolini and Straumann, 2016; Chen et al., 2016; Yakushin et al., 2017). The commonly invoked sensory mismatch theory posits that the visual and vestibular/somatosensory signals conflict, or in the absence of the visual inputs vestibular signals from otolith organs and semicircular canals mismatch to elicit the MS (Reason, 1978; Bles, 1998; Bles et al., 1998; Golding, 2006; Shupak and Gordon, 2006; Schmal, 2013; Oman and Cullen, 2014; Koch et al., 2018).

In the present study, we disposed rat nose-down, with its anteroposteiror axis tilting at an angle of 30°, rendering otolith organs receptive to unusual stimulus from gravitoinertial vector. As such, the signals from semicircular canals and otolith organs were presumably rearranged in a new fashion unlike that established through previous experience, and caused mismatch in the brain and thus the MS. Furthermore, in the frame of reference of the large rotating turntable, Coriolis force might possibly contribute to the linear-angular interaction (Holly, 2004). It is worthy to note that through the present simulating mode, the physical restraint of rat and the blockade of visual cues provided a physiological context deprived of visual stimulation with well-controlled proprioception. Therefore, taken together with the observation that VNC neurons were activated (Fos-IR neurons), it is reasonably to presume that the present rotation-induced MS was causally of vestibular origin. In fact, one of the most conventional approaches to activating vestibular end organs utilized linear acceleration and off-vertical rotation (Lai et al., 2004, 2010; Ma et al., 2013; Shimizu et al., 2015).

The centrifuges and the variant rotators were reportedly employed to elicit distinct forms of the MS including the visually induced MS and space MS (Lackner and Dizio, 2006; Keshavarz et al., 2015; Nooij et al., 2017). Our present MS-provoking rotator and setup differed not only from those with earth-horizontal axis (or axes), and from those with vertical axes as well (Takeda et al., 1986; Idoux et al., 2018). Notably, we eliminated the visual inputs and had the somatosensory inputs well-controlled. In this way, the contingency of cross coupling (head tilt while rotating) (Lackner and Graybiel, 1986; Romano et al., 2017) or unstable proprioceptive inputs (body movements) that are unpredictable, aleatory variables for different individuals might be minimized. Therefore, the present stimulating mode may technically render experimental results more comparable between individual animals or different experiment batches. In contrast, most previous studies reported rodent MS inducement by employing relatively large cages, rendering mobility possible and admitting light signals (Takeda et al., 1986, 1989; Li et al., 2005; Xiaocheng et al., 2012; Inprasit et al., 2018; Machuca-Márquez et al., 2021). Under these circumstances, the strength of the proprioceptive input may change from one moment to another during stimulation. Thus, it is very likely to generate, in separate stimulating sessions, the results from the animal(s) receiving signal inputs with differential strength or from the different combinations of vestibular, visual and proprioceptive receptors.

### Deliberation on the Statistic Processing of Data on Pica

In addition to pica, the useful ethological surrogates indexing rodent MS include also CTA (Ossenkopp and Tu, 1984; Fox, 1990; Andrews and Horn, 2006; Xiaocheng et al., 2012), piloerection, grooming, urination and fecal incontinence (Yu et al., 2007; Wei et al., 2011), and neurohypophyseal hormones such as vasopressin and oxytocin (Li et al., 2005). However, pica is the most frequently invoked classic agent in the assessment of rodent MS (Mitchell et al., 1977; Takeda et al., 1989; Santucci et al., 2000; Schmal, 2013; Idoux et al., 2018; Inprasit et al., 2018; Nakajima, 2018), with the advantage of possessing no subjectivity. Therefore, an unbiased data processing on pica insusceptible to possible misinterpretation of experimental results in relation to MS is of great importance.

We took two sets of pica data from Phase 3 as the variables: (1) the gross values representing individual’s total amount of KI, i.e., the logged daily KI, and (2) net increased amount (or gain) in daily KI, represented arithmetically by subtracting baseline from the four-day average of daily KI post rotation (Equation 2 in “Materials and Methods”). The hypothesis tests showed that using both gross and gain values yielded the following consistent outcome: A significant increase of KI (g/day) after rotary stimulation (Figures 4F,H). However, a caveat concerning the use of the gross values should be made.

Previous work utilized the gross values of the daily KI for assessing rodent MS (Takeda et al., 1986; Horii et al., 1993; Idoux et al., 2018; Inprasit et al., 2018). It is worthy to note that the gross value implicates at least two of the following biological components: (1) The gain of KI in close relation to individual’s susceptibility to MS-provocation and (2) the interindividual difference in baseline KI. Obviously, it is the gain or net increase of KI per day that exactly concerns MS. Therefore, the gross value, as compared with gain in KI, probably less truthfully characterized MS.

In the present study, we observed that some naive rats normally ate kaolin (daily KI > 0. 5 g/per day) even after habituation phase (Figures 2A, 4A,B and Table 1). The amount of daily KI differed between rats at the same day, and fluctuated with variability from day to day as well. This phenomenon was *de facto* repetitively documented (Santucci et al., 2000; Idoux et al., 2018; Inprasit et al., 2018; Nakajima, 2018). Therefore, the different rats showed variability in KI baseline levels. As a result, if gross daily KI would be “jumbled” in an experimental group while performing hypothesis tests, the interindividual distinctions in MS susceptibility might be masked. The reason is that the gross KI was not always in proportion to MS score (or gain in daily KI). This could be readily exemplified by rats 6# *vs.* 7# and rats 4# *vs.* 7# in Table 2, revealing that the similar gross amount of daily KI following the rotation did not necessarily implicate similar gain in daily KI, and *vice versa*. Specifically, the rats 6# and 7# were similar in the gross daily KI, but their respective gains were set far apart; rats 4# *vs.* 7# approximated in gains but differed greatly in the gross daily KI (Table 2). Based on this analysis, we suggested taking net instead of gross values of KI as independent variables in hypothesis testing would be more appropriate. This is especially necessary when non-paired hypothesis testing is performed between the two differently treated groups of animals, e.g., between two groups receiving different prospective anti-emetic drugs, where the two groups may have differential baseline KI levels due to drug action.

Noteworthily, notwithstanding a significant difference between pseudo- and rotation-stimulation groups was observed when the gains in KI per day (g/day) over either 1-, 2-, 3-, or 4-day period following the rotation were used (see “Results”), two causes prompted us to utilize the average gain of daily KI over 4-day (but not 1-, 2- or 3-day) period for data processing. First, we observed that, in general, daily KI peaked at day 1 after the rotation and then gradually decreased over time; however, for some animals, relatively higher than baseline level regarding KI was observed after day 1, day 2 or even day 3 in Phase 3. The earlier reports on mice MS study was in consistence with this phenomena (Santucci et al., 2000, 2009). This may be due to the “lingering” pica behavior that trailed rotary stimulation, or to the inherent fluctuating property of pica in rodents. Further study is still required to address this issue. Second, choosing a 4-day time block to evaluate the pica in Post-R session was consistent with that used for baseline estimation in Phase 1.

For the further understanding, we pose here a couple of questions: Why should not take a single-day time period? Imagine if the daily KI at any single day was employed as variables for scoring the pica behavior, which day was the appropriate candidate in the context of dynamic daily KI change from day to day? It is reasonable to infer that using average daily KI over a longer period which includes as many days witnessing higher Post-R KI level as possible might be more truthful in characterizing pica for each rat.

## Conclusion

The setup in this study that was employing double-axis rotation to stimulate vestibular apparatus was effective in provoking the MS in rat; it may serve as a useful tool targeting at dissection of the neural substrates in connection with the vestibular activity that provokes the MS, and also as a tool for the anti-MS drug screening by supplying animal-based psychopharmacological data from lab work.

## Data Availability Statement

The original contributions presented in the study are included in the article/supplementary material, further inquiries can be directed to the corresponding authors.

## Ethics Statement

The animal study was reviewed and approved by Committee on the Animal Experimental Welfare and Ethics, The Fourth Military Medical University.

## Author Contributions

F-XZ, Y-QL, and HL designed the rotary setup and experiment protocols, drafted the manuscript, conceptualized the interpretation, and analyzed the data. Z-HZ, L-PL, YF, X-CW, and WW conducted the experiments and collected and compiled the data. Y-SC and LW participated in the experiment design and text revision. All authors contributed to the article and approved the submitted version.

## Conflict of Interest

The authors declare that the research was conducted in the absence of any commercial or financial relationships that could be construed as a potential conflict of interest.

## Publisher’s Note

All claims expressed in this article are solely those of the authors and do not necessarily represent those of their affiliated organizations, or those of the publisher, the editors and the reviewers. Any product that may be evaluated in this article, or claim that may be made by its manufacturer, is not guaranteed or endorsed by the publisher.

## Abbreviations

CTA: conditioned taste avoidance
IR: immunoreactivity
KI: kaolin intake
MS: motion sickness
MVe: medial vestibular nucleus
OFT: open field test
Pr: prepositus nucleus
Pre-R: pre-rotation
Post-R: post-rotation
SpVe: spinal vestibular nucleus
VNC: vestibular nucleus complex.
